# HTLV-1 Japanese subgroup in Brazil: phylogenetic and migratory history

**DOI:** 10.1186/s12977-025-00663-4

**Published:** 2025-05-02

**Authors:** Carolina Amianti, Larissa Melo Bandeira, Aline Pedroso Lorenz, Tayana Serpa Ortiz Tanaka, João Américo Domingos, Ana Rita Coimbra Motta-Castro

**Affiliations:** 1 Laboratório de Imunologia Clínica, Faculdade de Ciências Farmacêuticas, Alimentos e Nutrição, Universidade Federal de Mato Grosso do Sul, Campo Grande, MS Brazil; 2 Laboratório de Ecologia e Biologia Evolutiva, Instituto de Biociências, Universidade Federal de Mato Grosso do Sul, Campo Grande, MS Brazil; 3 Instituto de Análises Laboratoriais Forenses, Coordenadoria-Geral de Perícias de Mato Grosso do Sul, Campo Grande, MS Brazil; 4 Faculdade de Medicina, Universidade Federal de Mato Grosso do Sul, Campo Grande, MS Brazil

**Keywords:** Molecular clock, HTLV-1aJpn, Japan, Brazil

## Abstract

**Background:**

The retrovirus Human T-lymphotropic virus type 1 is classified into different subtypes, and due to its low evolutionary rates, it can be used to explore geographic patterns of origin and dispersion of human populations. In Brazil, Transcontinental and Japanese subgroups, from the Cosmopolitan subtype, are the more common lineages, with prevalence rates notably higher among Japanese immigrants and their descendants. The study aimed to trace the history and circulation of the Japanese subgroup in Brazil using phylogenetic and populational analyses.

**Methods:**

A total of 381 HTLV-1 long terminal repeat region sequences were retrieved from the GenBank database. Phylogenetic and molecular clock analysis were performed using Maximum Likelihood and Bayesian Inference methods. A median-joining network was constructed to assess the relationships among the haplotypes of the Japanese subgroup.

**Results:**

This study found that the HTLV-1 LTR sequences from Japanese immigrants and their descendants in Brazil formed two major clades, Transcontinental (HTLV-1aTC) and Japanese (HTLV-1aJpn). Seventy-four haplotypes were identified in the haplotype network and the estimate of Japanese clade divergence dates 18,748 years ago (95% CI13,348 to 24,767 years).

**Conclusion:**

Our study corroborates the recent migratory movements as the potential mechanism for HTLV-1aJpn introduction in Brazil.

**Supplementary Information:**

The online version contains supplementary material available at 10.1186/s12977-025-00663-4.

## Introduction

Human T-lymphotropic virus 1 (HTLV-1) is a neglected infection that is associated with two severe clinical outcomes: HTLV-1 Associated Myelopathy (HAM) and Adult T-cell Leukemia/Lymphoma (ATL) [1]. The virus is globally distributed, with high endemic levels in some regions of Southern Japan, native Aboriginal Australian, Central and South Africa, the Caribbean Islands, Melanesia, and Central and South America [2–6].

The HTLV-1 genome shows remarkable genetic stability; however, the long terminal repeat (LTR) region displays considerable diversity, rendering it an optimal target for phylogenetic studies. This diversity allows the classification of HTLV-1 into distinct subtypes (from 1-a to 1-g) and subgroups. Sequences based on this region of the HTLV-1 genome are available in databases from various countries [7]. The Cosmopolitan genotype (HTLV-1a) is classified into six subgroups (A to F) that are geographically related and show the most extensive distribution in the world, present in all continents [5, 8–14].

In Japan, both subgroups, Transcontinental (HTLV-1aTC) and Japanese (HTLV-1aJpn), have been identified in different geographical regions. HTLV-1 infection prevails among Ainu, Ryukyuans and Wajin, three ethically distinguishable Japanese populations that live in the northern (Hokkaido), southern (Okinawa) and central (Honshu, Shikoko, and Kyushu) islands, respectively. HTLV-1aTC subgroup was introduced more recently in Japan, and it is mostly found in the North and Southwest islands, Hokkaido and Okinawa, respectively. While the HTLV-1aJpn subgroup is predominant among the Wajin, the mainland Japanese population [5, 14–18].

The prevailing hypothesis of the introduction of HTLV-1 in South America dates back to two different movements with different timing: from Africa through the African slave trade during the colonial era and, Japanese migration in the twentieth century [6, 12, 19–21]. In Brazil, both Transcontinental (aTC) and Japanese (aJpn) subgroups have been identified [6, 22–26].

Japanese immigration to Brazil began in 1908 with the arrival of the Kasato Maru ship, due to Brazil’s demand after the abolition of slavery and Japan’s economic challenges, furthermore, other flows during pre and post-World War II., which made Brazil the largest Nikkei community outside Japan, with up to 1,22 million individuals by the late twentieth century [27, 28].

Brazilian population presents a mixture of races and cultures, due to the historical formation that mainly includes descendants from European colonizers, African slaves, native Indians, and immigrants from Asia [29].

Approximately 2 million Japanese descendants reside in Brazil [30]. The large presence of this population group could explain the prevalence of HTLV-1aTC and HTLV-1aJpn subgroups. Although the HTLV-1 infection is endemic in South America with the considerable frequency of HTLV-1aJpn circulating in Brazil among Japanese immigrants and their descendants [25, 26], there is scarce information about the history of the introduction of HTLV-1aJpn in our country. Therefore, this investigation was conducted to address this knowledge gap about the HTLV-1aJpn subgroup and to get a clear idea about the origin of these isolates in Brazil. Analyze the history and circulation of the Cosmopolitan subtype (1a), the Japanese subgroup in Brazil using all available sequences of this subgroup.

## Materials and methods

### Sampling

The database includes information from 111 HTLV-positive Japanese immigrants and their descendants from Central and Southeast Brazil, based on data from previous studies that have already been published. The participants were also interviewed about their historical characteristics [25, 26]. The HTLV-1 LTR dataset generated in these studies comprised 18 sequences from individuals living in Campo Grande (Mato Grosso do Sul state) and 93 sequences from individuals residing in São Paulo (São Paulo state). Among these, 32 sequences were from Japanese immigrants, mainly from Okinawa, and 78 were from Japanese descendants born in the Brazilian states of São Paulo, Mato Grosso do Sul, or Paraná. Furthermore, one sequence was obtained from a Japanese descendant from Peru living in Campo Grande, MS (KM023764—OKW72).

To expand the initial dataset, additional sequences were obtained from the GenBank database using the following keywords: *HTLV* (AND) *LTR* (AND) *Japanese* (OR) *Japan*. Reference sequences for the Japanese and Transcontinental HTLV subtypes were selected based on Afonso et al. [14]. All sequences identified through this search were verified using BLAST, and the top 10 match results, based on coverage and pairwise identity, were incorporated into the dataset. To ensure data quality and comparability, sequences without information about geographical origin or shorter than 400 bp were excluded from further analysis. The final dataset, comprising all publicy available sequences from Japanese subgroup in GenBank, includes a total of 381 sequences. Details, such as GenBank accession numbers, are provided in Supplementary Spreadsheet 1.

Sequences from the Transcontinental clade were subsequently subjected to Blast, and the top 10 hits, based on coverage and pairwise identity, were select to ensure the highest similarity, due to the search keywords not providing all sequences about the Transcontinental group, resulting in only a partial result about of this lineage.

### Phylogenetic analyses

The sequence alignments were conducted using the MAFFT plugin, version 7.308, available within Geneious software, version 7.1.3, employing the FFT-NS-i algorithm [31]. A reference sequence, AF124043, was used to guide alignment for the LTR sequence region. The final alignment consisted of 775 base pairs. Nucleotide substitution models were selected using Bayesian Information Criteria (BIC) via the JModelTest online version using CIPRES Science Gateway [32].

Phylogenetic trees were reconstructed using Maximum Likelihood analysis (ML) and Bayesian Inference (BA). ML analyses were performed using RAxML [33], utilizing the GTRGAMMA model, with support values estimated from 1,000 bootstrap pseudoreplicates. For Bayesian analysis, we used the Beast 2.6.7 package [34], employing the uncorrelated lognormal relaxed molecular clock and the Birth and Death Model [35, 36]. We utilized the GTR + G substitution model with four gamma categories. To estimate the Japanese clade age (HTLV 1aJpn lineage), we employed a Bayesian evaluation using a relaxed clock model with an uncorrelated lognormal distribution of substitution rates. The evolutionary rate for the LTR gene was derived from Lemey et al. [37] as 5.6 × 10^–7^ (ranging from 1.2 × 10^–7^ to 1.1 × 10^–6^) substitutions per site per year. Our analysis consisted of two independent Markov Monte Carlo Chains (MCMC) runs, each covering 100,000,000 generations and sampling every 10,000 generations, and the first 25% of generations were discarded as burn-in. JmodelTest, RAxML, and Beast analyses were performed on CIPRES portal. The Tracer program [38] was employed to assess the sampling convergence of the Bayesian analysis (BA) through the estimated effective sampling size (ESS). An ESS value exceeding 200 indicated convergence was achieved. The trees generated were edited using the Interactive Tree Of Life (iTOL) software v6 (https://itol.embl.de) [39].

The relationships within the Japanese subgroup (HTLV-1aJpn) were also inferred by constructing haplotype networks using the Neighbor-Joining algorithm, implemented in the PopArt software version 1.6 [40].

## Results

Our sampling of 111 HTLV-1-positive sequences from previous studies comprised 63 of the Japanese and 48 of the Transcontinental subgroup. The sequences obtained from the GenBank search underwent an exploratory analysis to determine their lineage. After excluding repeated sequences, the final dataset comprised 138 LTR sequences of the Transcontinental subgroup and 220 sequences of the Japanese subgroup.

**Fig. 1:**
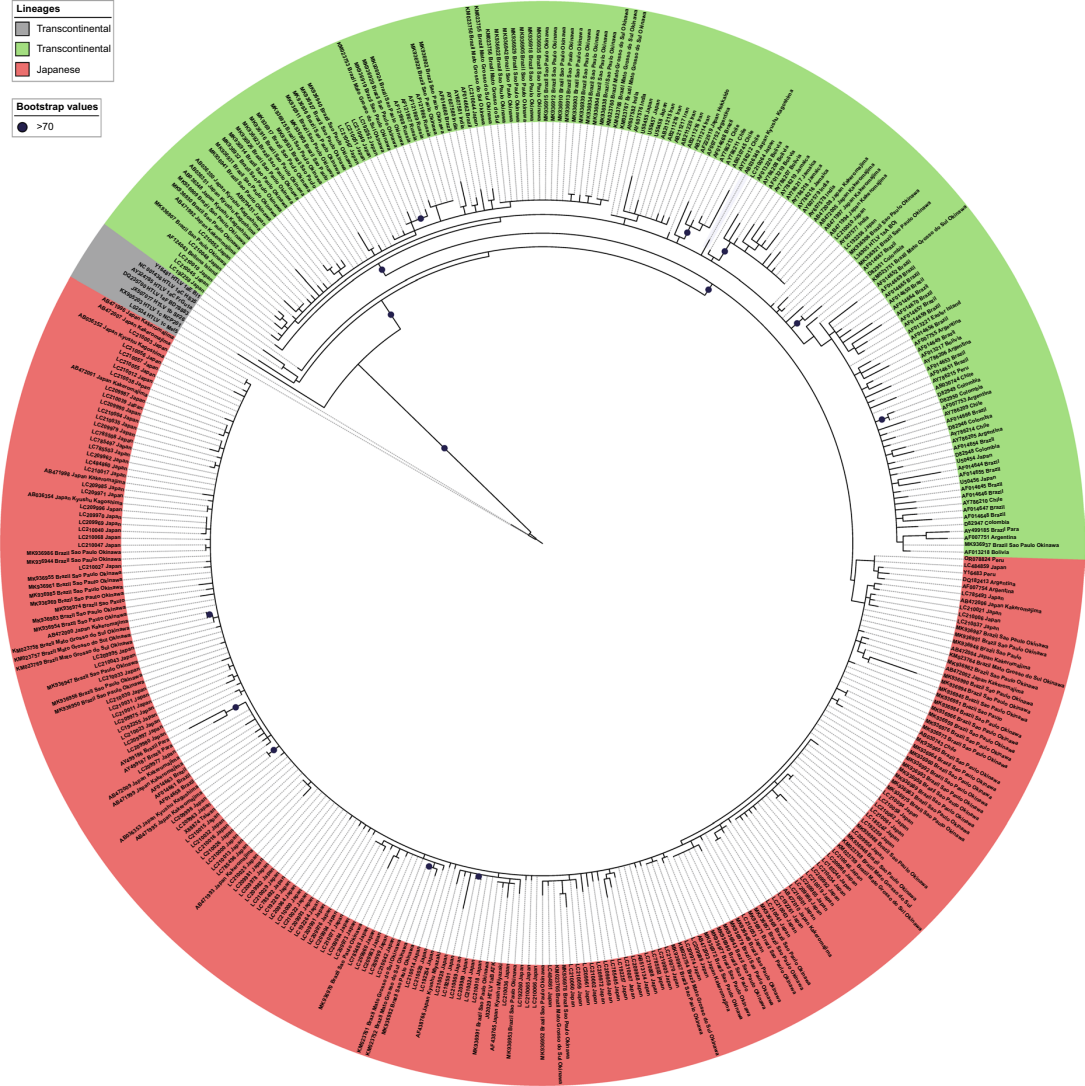
Maximum Likelihood tree constructed using LTR sequences of the Transcontinental and Japanese lineages. Bootstrap values > 50 are indicated at the nodes

The phylogenetic tree reconstructed using ML analysis (Fig. 1) recovered the two major clades corresponding to the Transcontinental (HTLV-1aTC) and Japanese (HTLV-1aJpn) lineages. The Transcontinental clade comprises sequences from individuals in Argentina (n = 6), Bolivia (n = 6), Brazil (n = 74), Chile (n = 8), Colombia (n = 6), India (n = 7), Iran (n = 6), Jamaica (n = 4), Japan (n = 29), Peru (n = 1), and Russia (n = 4). It is important to note that the sampling of the Transcontinental lineage is partial, as the primary focus of this study was the Japanese lineage, including Japanese immigrants and their descendants who live in Brazil.

**Fig. 2:**
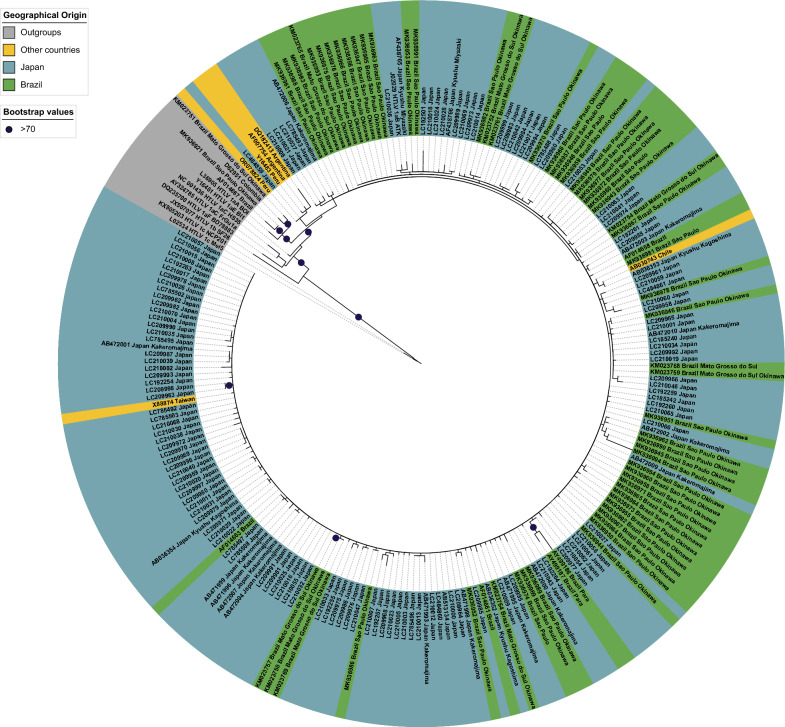
Maximum Likelihood tree constructed using LTR sequences of the Japanese subgroup, highlighting the geographical origin of the samples. Bootstrap values > 70 are indicated at the nodes

Among the LTR sequences of the Transcontinental lineage recovered in our search, 74 were obtained from Brazilian samples, and 24.32% showed a higher similarity (top 10 BLAST hits) with sequences from Brazil and Japan; 40.54% from Brazil and other countries including Japan; 17.57% with Japan and other countries, except Brazil; 5.41% from Brazil and other countries except Japan, and 12.16% only from Brazil. Therefore, 82.43% of the Transcontinental sequences from Japanese descendants living in Brazil have a close relationship with sequences from Japan. Figure 2 highlights that the 220 sequences of the Japanese (HTLV-1aJpn) lineage are primarily from Brazil and Japan but also from other countries, in which 67 sequences (30.45%) are Japanese or their descendants living in Brazil.

Transcontinental sequences (MK936921, AF014667, D82951, KM023751) were included in aim to stabilize the ML analysis and were highlighted as outgroups.

**Fig. 3:**
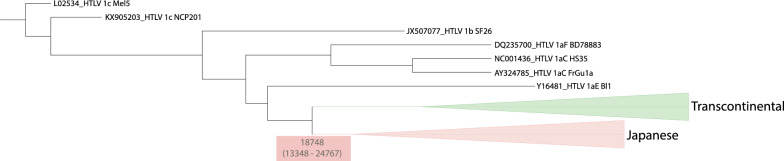
Maximum clade credibility tree of Transcontinental and Japanese lineages reconstructed in BEAST using the LTR dataset. The estimate of the Japanese clade divergence and the 95% highest posterior density intervals are indicated below the node

**Fig. 4:**
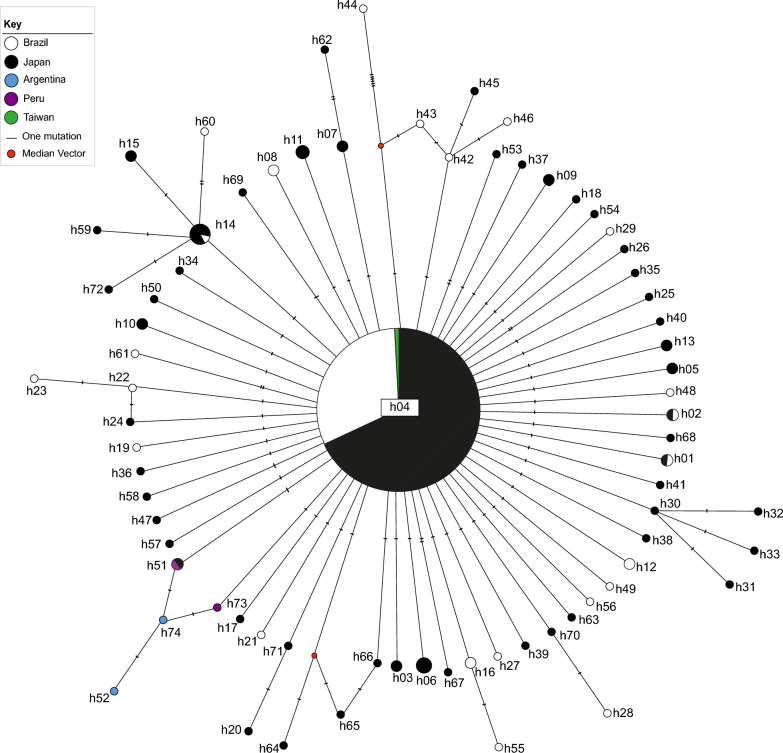
Haplotype median-joining network constructed with LTR sequences of the Japanese lineage detected in individuals of different countries. The size of the circles is proportional to the number of sequences with the same haplotype, and the colors represent the geographic origin

The maximum clade credibility tree reconstructed in BEAST estimated that the divergence of the most recent common ancestor (MRCA) of the Japanese lineage most likely occurred about 18.000 years ago (95% highest posterior density intervals: 13.348 – 24.767) (Fig. 3). The mixture of HTLV-1 virus within the Japanese clade (with sequences from Japan, Brazil, Argentina, Peru, and Taiwan), without distinct geographic groups, reinforces the hypothesis that the introduction of the virus occurred through Japanese immigration from the early twentieth century. Among the 220 sequences of the Japanese subgroup, 74 haplotypes were identified, displaying shallow divergence. This pattern is particularly evident in the starlike network (Fig. 4), where a central haplotype (h04) exhibits a high frequency and wide geographical distribution. The network indicates that h04 is the ancestral haplotype, and most other haplotypes branch from it. This likely ancestral haplotype coexists with the derived haplotypes, lacking phylogeographic subdivisions in their relationship.

## Discussion

The history of Japanese immigration to Brazil is tied to the broader historical context of the Ryukyu Kingdom and Okinawa. The Ryukyu Kingdom, existed from 1429 until it was annexed by Japan in 1879, transforming into Okinawa Prefecture. Although became a part of Japan, it retained distinct cultural, linguistic, and historical differences 41, 42].

The formal relationship between Brazil and Japan was established in 1895 by the “Treaty of Friendship, Commerce and Navigation”, and in 1908, especially for the cultivation of coffee plantations after the abolition of slavery, the first wave of Japanese immigrants, including 325 Okinawans, arrived in São Paulo. In 1914, Okinawans settled in Campo Grande (MS), and by 1952, Brazil had become home to 229,000 Japanese immigrants [41, 42]. Despite confronted harsh conditions in coffee plantations in São Paulo and in the construction of the Noroeste do Brasil Railway, they made significant contributions to Brazil’s cultural and economic development [41, 42].

Currently, approximately 2 million Japanese descendants reside in Brazil, the largest community outside Japan, primarily concentrated in São Paulo (Southeast region), Mato Grosso do Sul (Central-West region), Paraná (South region), and Pará (North) states [30, 43]. Among the health challenges of in this population is an above-average incidence of people infected with the HTLV-1 virus. In Brazil, the first detection of HTLV-1 infection was identified among Japanese immigrants in Campo Grande, Mato Grosso do Sul, in 1986, with a prevalence of 10% [21]. Several studies conducted by our group among Japanese immigrants and their descendants found a high prevalence of HTLV-1 infection of 6.8% in Campo Grande, MS, and 5.1% in São Paulo [25, 26].

The HTLV-1 infection is endemic in some regions of Japan, and its distribution has a heterogeneous pattern, with a high prevalence in the Southwestern region, specifically in the Kyushu and Okinawa islands and in the North in Hokkaido, between indigenous population [4, 44]. The Transcontinental subgroup was initially introduced, followed by a second wave of migration introducing the Japanese subgroup [44]. Our data demonstrate that, after the introduction of these lineages into Brazil, the short period of time and the low mutation rate did not allow for the differentiation of specific clades. The Japanese and Transcontinental sequences obtained from our study, brought by Japanese immigrants to Brazil in the twentieth century, correspond to these prehistoric lineages from Japan.

A deeper look at the haplotypic variation patterns of the Japanese lineage revealed a typical star-like network (Fig. 4), where a central haplotype occurs in many individuals and is geographically widespread. This ancestral haplotype probably gave rise to several other minimally divergent haplotypes in a process of recent population expansion. The recent history of Japanese migration to Brazil [27] supports this finding. There has been insufficient time for the Japanese sequences from Japan and Brazil to differentiate, given the low evolutionary rate of HTLV-1.

The median-joining network and the time of divergence for the Japanese clade provide complementary information among the Japanese subgroup sequences, an interesting fact since the two analysis tools do not always generate congruent results [45]. The estimated time of divergence for the Japanese clade is approximately 18,000 years ago, and there is no geographical differentiation between the sequences from residents of Japan and the descendants of immigrants. The prehistoric origin of the lineage, combined with a low mutation rate and a recent population expansion, do not allow us to infer exactly which haplotypes came from Japan or when the exclusive haplotypes in Brazil emerged.

The current study represents the most extensive evolutionary analysis of HTLV-1aJpn infection in Brazil that has been conducted until now. However, some limitations must be acknowledged, such as the presence of a sampling bias, as the majority of the available sequences originated from Brazil and Japan may have influenced the study's findings. Furthermore, sequences accessible in GenBank were not included in our dataset due to the absence of geographic origin data, It is highly recommended that, during the inclusion of sequences in GenBank, researchers provide complete metadata to maximize the utility of available data in future studies. Furthermore, expanding future studies to include sequences from other South American countries with significant Japanese immigrant populations should be considered.

This study is the first in Brazil to extensively analyze the evolution of HTLV-1aJpn in the major Japanese population outside Japan. The presence of mixed viral sequences between Japanese and Brazilians, along with historical data, points to the recent migratory movements as responsible for the introduction of this lineage in Brazil. Given the significant prevalence of HTLV-1 within certain demographic groups, it is important to enhance epidemiological surveillance and implement screening strategies that consider historical and cultural factors. Essential actions include systematic HTLV-1 screening, coupled with culturally appropriate counseling and educational programs aimed at preventing sexual and intrafamilial transmission. These measures are crucial for improving public health policies and controlling HTLV-1 in diverse communities.

## Supplementary Information

Supplementary material 1.

Supplementary material 2.

## Data Availability

No datasets were generated or analysed during the current study.

## References

[1] Ramesh N, Cockbain B, Taylor GP, Rosadas C. How do socioeconomic determinants of health affect the likelihood of living with HTLV-1 globally? A systematic review with meta-analysis. Front Public Health. 2024;12:1298308. 10.3389/fpubh.2024.1298308. 38327581 10.3389/fpubh.2024.1298308 PMC10848500

[2] Catovsky D, et al. Adult T-cell lymphoma-leukaemia in Blacks from the West Indies. Lancet. 1982;1(8273):639–43. 10.1016/s0140-6736(82)92200-0. 6121963 10.1016/s0140-6736(82)92200-0

[3] Schrijvers D, Delaporte E, Peeters M, Dupont A, Meheus A. Seroprevalence of retroviral infection in women with different fertility statuses in Gabon, western equatorial Africa. J Acquir Immune Defic Syndr. 1991;4(5):468–70. 2016684

[4] Watanabe T. Current status of HTLV-1 infection. Int J Hematol. 2011;94(5):430–4. 10.1007/s12185-011-0934-4. 21969187 10.1007/s12185-011-0934-4

[5] Gessain A, Cassar O. Epidemiological aspects and world distribution of HTLV-1 infection. Front Microbiol. 2012;3:388. 10.3389/fmicb.2012.00388. 23162541 10.3389/fmicb.2012.00388 PMC3498738

[6] Paiva A, Casseb J. Origin and prevalence of human T-lymphotropic virus type 1 (HTLV-1) and type 2 (HTLV-2) among indigenous populations in the Americas. Rev Inst Med Trop Sao Paulo. 2015;57(1):1–13. 10.1590/S0036-46652015000100001. 25651320 10.1590/S0036-46652015000100001 PMC4325517

[7] Pourrezaei S, et al. Molecular epidemiology and phylogenetic analysis of human T-lymphotropic virus type 1 in the tax gene and it association with adult t-cell leukemia/lymphoma disorders. Iran J Microbiol. 2021;13(4):509–17. 10.1850/ijm.v13i4.6976. 34557280 10.18502/ijm.v13i4.6976 PMC8421578

[8] Seiki M, Hattori S, Hirayama Y, Yoshida M. Human adult T-cell leukemia virus: complete nucleotide sequence of the provirus genome integrated in leukemia cell DNA. Proc Natl Acad Sci USA. 1983;80(12):3618–22. 10.1073/pnas.80.12.3618. 6304725 10.1073/pnas.80.12.3618 PMC394101

[9] Malik KT, Even J, Karpas A. Molecular cloning and complete nucleotide sequence of an adult T cell leukaemia virus/human T cell leukaemia virus type I (ATLV/HTLV-I) isolate of Caribbean origin: relationship to other members of the ATLV/HTLV-I subgroup. J Gen Virol. 1988;69(Pt 7):1695–710. 10.1099/0022-1317-69-7-1695. 2899128 10.1099/0022-1317-69-7-1695

[10] Gasmi M, Farouqi B, d’Incan M, Desgranges C. Long terminal repeat sequence analysis of HTLV type I molecular variants identified in four north African patients. AIDS Res Hum Retrovir. 1994;10(10):1313–5. 10.1089/aid.1994.10.1313. 7848687 10.1089/aid.1994.10.1313

[11] Bazarbachi A, et al. Human T-cell-leukemia virus type I in post-transfusional spastic paraparesis: complete proviral sequence from uncultured blood cells. Int J Cancer. 1995;63(4):494–9. 10.1002/ijc.2910630406. 7591256 10.1002/ijc.2910630406

[12] Van Dooren S, et al. Evidence for a post-Columbian introduction of human T-cell lymphotropic virus [type I] [corrected] in Latin America. J Gen Virol. 1998;79(Pt 11):2695–708. 10.1099/0022-1317-79-11-2695. 9820145 10.1099/0022-1317-79-11-2695

[13] Treviño A, et al. Molecular epidemiology and clinical features of human T cell lymphotropic virus type 1 infection in Spain. AIDS Res Hum Retrovir. 2014;30(9):856–62. 10.1089/AID.2013.0128. 24924996 10.1089/aid.2013.0128 PMC4151066

[14] Afonso PV, Cassar O, Gessain A. Molecular epidemiology, genetic variability and evolution of HTLV-1 with special emphasis on African genotypes. Retrovirology. 2019;16(1):39. 10.1186/s12977-019-0504-z. 31842895 10.1186/s12977-019-0504-z PMC6916231

[15] Vidal AU, et al. Molecular epidemiology of HTLV type I in Japan: evidence for two distinct ancestral lineages with a particular geographical distribution. AIDS Res Hum Retrovir. 1994;10(11):1557–66. 10.1089/aid.1994.10.1557. 7888210 10.1089/aid.1994.10.1557

[16] Yamashita M, Ishida T, Ohkura S, Miura T, Hayami M. Phylogenetic characterization of a new HTLV type 1 from the Ainu in Japan. AIDS Res Hum Retrovir. 2001;17(8):783–7. 10.1089/088922201750237068. 11429119 10.1089/088922201750237068

[17] Otani M, et al. Distribution of two subgroups of human T-lymphotropic virus type 1 (HTLV-1) in endemic Japan. Trop Med Health. 2012;40(2):55–8. 10.2149/tmh.2012-02. 23097620 10.2149/tmh.2012-02 PMC3475314

[18] Otani M, et al. Phylogeography of human T-lymphotropic virus type 1 (HTLV-1) lineages endemic to Japan. Trop Med Health. 2012;40(4):117–24. 10.2149/tmh.2012-15. 23532551 10.2149/tmh.2012-15 PMC3598069

[19] Li HC, et al. The presence of ancient human T-cell lymphotropic virus type I provirus DNA in an Andean mummy. Nat Med. 1999;5(12):1428–32. 10.1038/71006. 10581088 10.1038/71006

[20] Alcantara LC, et al. Globin haplotypes of human T-cell lymphotropic virus type I-infected individuals in Salvador, Bahia, Brazil, suggest a post-Columbian African origin of this virus. J Acquir Immun Defic Syndr. 2003;33(4):536–42. 10.1097/00126334-200308010-00016. 10.1097/00126334-200308010-00016 12869844

[21] Kitagawa T, Fujishita M, Taguchi H, Miyoshi I, Tadokoro H. Antibodies to HTLV-I in Japanese immigrants in Brazil. JAMA. 1986;256(17):2342. 2877100

[22] Vallinoto AC, et al. Serological and molecular evidence of HTLV-I infection among Japanese immigrants living in the Amazon region of Brazil. Jpn J Infect Dis. 2004;57(4):156–9. 15329447

[23] Souza LA, et al. Caracterização molecular do HTLV-1 em pacientes com paraparesia espástica tropical/mielopatia associada ao HTLV-1 em Belém, Pará [Molecular characterization of HTLV-1 among patients with tropical spastic paraparesis/HTLV-1 associated myelopathy in Belém, Pará]. Rev Soc Bras Med Trop. 2006;39(5):504–6. 10.1590/s0037-86822006000500017. 17160333 10.1590/s0037-86822006000500017

[24] Galvão-Castro B, et al. Epidemiologia e origem do HTLV-1 em Salvador Estado da Bahia: a cidade com a mais elevada prevalência desta infecção no Brasil. Gazeta Médica da Bahia. 2009;79:3–10.

[25] Bandeira LM, et al. High prevalence of HTLV-1 infection among Japanese immigrants in non-endemic area of Brazil. PLoS Negl Trop Dis. 2015;9(4): e0003691. 10.1371/journal.pntd.0003691. 25886507 10.1371/journal.pntd.0003691 PMC4401538

[26] Bandeira LM, et al. Human T-cell leukemia virus type 1 infection among Japanese immigrants and their descendants living in Southeast Brazil: a call for preventive and control responses. PLoS Negl Trop Dis. 2021;15(2): e0009066. 10.1371/journal.pntd.0009066. 33544713 10.1371/journal.pntd.0009066 PMC7864455

[27] Miyasaka LS, et al. Mental health of two communities of Japanese-Brazilians: a comparative study in Japan and in Brazil. Psychiatry Clin Neurosci. 2002;56(1):55–64. 10.1046/j.1440-1819.2002.00929.x. 11929571 10.1046/j.1440-1819.2002.00929.x

[28] Sasaki E. A imigração para o Japão. Estud av. 2006;57:99–117. 10.1590/S0103-40142006000200009.

[29] Motta-Castro AR, et al. Hepatitis B virus infection in isolated Afro-Brazilian communities. J Med Virol. 2005;77(2):188–93. 10.1002/jmv.20435. 16121385 10.1002/jmv.20435

[30] BRASIL. Ministério do Turismo. 114 anos de Japão no Brasil. 2022. https://www.gov.br/turismo/pt-br/assuntos/noticias/114-anos-de-japao-no-brasil

[31] Katoh K, Misawa K, Kuma K, Miyata T. MAFFT: a novel method for rapid multiple sequence alignment based on fast Fourier transform. Nucleic Acids Res. 2002;30(14):3059–66. 10.1093/nar/gkf436. 12136088 10.1093/nar/gkf436 PMC135756

[32] Miller M. A.,. Pfeiffer W &. Schwartz T. Creating the CIPRES Science Gateway for inference of large phylogenetic trees. Gateway Computing Environments Workshop (GCE), New Orleans, LA, USA, 2010, pp. 1-8, 10.1109/GCE.2010.5676129

[33] Stamatakis A. RAxML version 8: a tool for phylogenetic analysis and post-analysis of large phylogenies. Bioinformatics. 2014;30(9):1312–3. 10.1093/bioinformatics/btu033. 24451623 10.1093/bioinformatics/btu033 PMC3998144

[34] Drummond AJ, Suchard MA, Xie D, Rambaut A. Bayesian phylogenetics with BEAUti and the BEAST 17. Mol Biol Evol. 2012;29(8):1969–73. 10.1093/molbev/mss075. 22367748 10.1093/molbev/mss075 PMC3408070

[35] Drummond AJ, Ho SY, Phillips MJ, Rambaut A. Relaxed phylogenetics and dating with confidence. PLoS Biol. 2006;4(5): e88. 10.1371/journal.pbio.0040088. 16683862 10.1371/journal.pbio.0040088 PMC1395354

[36] Stadler T. On incomplete sampling under birth-death models and connections to the sampling-based coalescent. J Theor Biol. 2009;261(1):58–66. 10.1016/j.jtbi.2009.07.018. 19631666 10.1016/j.jtbi.2009.07.018

[37] Lemey P, Pybus OG, Van Dooren S, Vandamme AM. A Bayesian statistical analysis of human T-cell lymphotropic virus evolutionary rates. Infect Genet Evol. 2005;5(3):291–8. 10.1016/j.meegid.2004.04.005. 15737921 10.1016/j.meegid.2004.04.005

[38] Rambaut A, Drummond AJ, Xie D, Baele G, Suchard MA. Posterior summarization in bayesian phylogenetics using tracer 17. Syst Biol. 2018;67(5):901–4. 10.1093/sysbio/syy032. 29718447 10.1093/sysbio/syy032 PMC6101584

[39] Letunic I, Bork P. Interactive tree of life (iTOL) v5: an online tool for phylogenetic tree display and annotation. Nucleic Acids Res. 2021;49(W1):W293–6. 10.1093/nar/gkab301. 33885785 10.1093/nar/gkab301 PMC8265157

[40] Leigh JW, Bryant D. POPART: full-feature software for haplotype network construction. Methods Ecol Evol. 2015;6:1110–6. 10.1111/2041-210X.12410.

[41] Associação Okinawa de Campo Grande - MS. Terra de Esperança Kibo no Daitsi 希望の大地. Campo Grande, MS: Life Editora. ISBN 978–85–8150–237–3. 2019

[42] Imigração Japonesa no Brasil http://www.imigracaojaponesa.com.br/.

[43] Embaixada do Japão no Brasil. Relações bilaterais entre Japão e Brasil. São Paulo: Embaixada do Japão no Brasil. https://www.sp.br.emb-japan.go.jp/pt/bilateral/embaixada.htm#

[44] Eguchi K, Fujii H, Oshima K, Otani M, Matsuo T, Yamamoto T. Human T-lymphotropic virus type 1 (HTLV-1) genetic typing in Kakeroma Island, an island at the crossroads of the ryukyuans and Wajin in Japan, providing further insights into the origin of the virus in Japan. J Med Virol. 2009;81(8):1450–6. 10.1002/jmv.21540. 19551824 10.1002/jmv.21540

[45] Kong S, Sánchez-Pacheco SJ, Murphy R. Median-joining networks and Bayesian phylogenies often do not tell the same story. Bull Soc Syst Biol. 2023. 10.18061/bssb.v2i1.9625.

