# Correction: HTLV-1 Japanese subgroup in Brazil: phylogenetic and migratory history

**DOI:** 10.1186/s12977-025-00666-1

**Published:** 2025-06-04

**Authors:** Carolina Amianti, Larissa Melo Bandeira, Aline Pedroso Lorenz, Tayana Serpa Ortiz Tanaka, João Américo Domingos, Ana Rita Coimbra Motta-Castro

**Affiliations:** 1 Laboratório de Imunologia Clínica, Faculdade de Ciências Farmacêuticas, Alimentos e Nutrição, Universidade Federal de Mato Grosso do Sul, Campo Grande, MS Brazil; 2 Laboratório de Ecologia e Biologia Evolutiva, Instituto de Biociências, Universidade Federal de Mato Grosso do Sul, Campo Grande, MS Brazil; 3 Instituto de Análises Laboratoriais Forenses, Coordenadoria-Geral de Perícias de Mato Grosso do Sul, Campo Grande, MS Brazil; 4 Faculdade de Medicina, Universidade Federal de Mato Grosso do Sul, Campo Grande, MS Brazil

**Correction: Amianti et al. Retrovirology (2025) 22:8**

10.1186/s12977-025-00663-4

Following publication of the original article [1], we were notified of an error in the last author’s name.

Originally published name: Ana Rita Coimbra Motta de Castro.

Corrected name: Ana Rita Coimbra Motta-Castro.

The original article has been corrected.
